# The Combined Effects of Sr(II) and Poly(Acrylic Acid) on the Morphology of Calcite

**DOI:** 10.3390/ma12203339

**Published:** 2019-10-13

**Authors:** Hak Yeong Kim, Taewook Yang, Wansoo Huh, Young-Je Kwark, Yunsang Lee, Il Won Kim

**Affiliations:** 1 Department of Chemical Engineering, Soongsil University, Seoul 06978, Korea; hacc02@naver.com (H.Y.K.); taugi@ssu.ac.kr (T.Y.); wshuh@ssu.ac.kr (W.H.); 2 Department of Organic Materials and Fiber Engineering, Soongsil University, Seoul 06978, Korea; ykwark@ssu.ac.kr; 3 Department of Physics, Soongsil University, Seoul 06978, Korea; ylee@ssu.ac.kr

**Keywords:** calcium carbonate, calcite, morphology, strontium ion, poly(acrylic acid)

## Abstract

Biomineralization of calcium carbonate has interesting characteristics of intricate morphology formation with controlled crystal polymorphs. In particular, modification of calcite morphology with diverse additives has been the focus of many biomimetic and bioinspired studies. The possible role of strontium ions in enhancing the morphology-modifying ability of macromolecules was investigated. In the present study, concentrations of strontium ions were comparable to that in seawater, and anionic poly(acrylic acid) and cationic poly(ethylene imine) were used as model macromolecules. When strontium ions were combined with anionic poly(acrylic acid), new types of calcite surfaces, most likely {hk0}, appeared to drastically change the morphology of the crystals, which was not observed with cationic poly(ethylene imine). This behavior of strontium ions was quite similar to that of magnesium ions, which is intriguing because both ions are available from seawater to be utilized during biomineralization.

## 1. Introduction

The biological crystallization of calcium carbonate has been intensively studied for the exoskeletons and endoskeletons of diverse biological species. For example, the calcium carbonate crystals present in the exoskeletons (shells) of mollusks are distinctively different from the geological counterparts in that the crystal phases and morphologies are precisely controlled [1] (pp. 88–110). Biological species are utilizing specific phases of calcium carbonate; some examples in the exoskeletons of mollusks include vaterite in gastropods, aragonite in nautiluses, and calcite and aragonite in mussels [1] (pp. 88–110). In addition to the anhydrous crystal phases of calcium carbonate (vaterite, aragonite, and calcite in increasing order of stability), amorphous calcium carbonate for temporary storage has been reported in the Florida applesnail (*Pomacea paludosa*), for example [1] (pp. 88–110).

The morphological diversity of biominerals is perhaps more remarkable in that the characteristic manifestation of the crystallographically predominant faces is often completely unnoticeable. For example, mature coccoliths of coccolithophores are constructed with calcite but display complex shapes with hammer-headed extensions, which are quite different from the rhombohedral morphology usually present in the geological cases [2]. In addition, each spine of a sea urchin has a complicated internal structure with overall circular cross-sections divided into sectors connected by spongy septa, but surprisingly corresponds to a single crystal of calcite [3]. Still, the fractured surface of the spine shows conchoidal cleavage unrelated to the usual {104} cleavage planes of calcite [3].

The extraordinary controls observed in the biomineralization of calcium carbonate have been mainly attributed to organic biomacromolecules. The composite biomacromolecular structure of β-chitin fibrils, silk-fibroin-like proteins, and acidic macromolecules is the classic model that instigated various ensuing studies on biomimetic and bioinspired crystallization ([1] (pp. 25–49), [3,4], and [5] (pp. 51–72)). Another interesting hypothesis is based on protein hydrogels composed of biomineralization-involved proteins with three distinctive sequences: intrinsically disordered (structurally extended), amyloid-like (self-aggregating), and folded (enabling protein–protein interactions) domains [6]. The biological crystallization has been also argued to follow paths different from classical crystallization, involving mesoscale assembly and amorphous precursors, as a result of the biomacromolecular control [5] (pp. 73–177). Nanoclusters have been found in echinoderms as well as mollusk shells, where organic macromolecules undoubtedly play an important role during the formation process ([5] (pp. 73–177), [7,8,9]).

Our research group has been interested in the inorganic ions, such as Mg(II), that enhance the crystallization-regulating ability of organic macromolecules. The enhancing ability of Mg(II) was confirmed for the prismatic-associated Asprich sequences (from the mollusk *Atrina rigida*), modulating the morphology of calcite to generate atypical {hk0} faces [10]. This behavior was also mimicked by the combination of Mg(II) and the synthetic macromolecules of poly(acrylic acid) and poly(methacrylic acid) [11]. In the present study, we examined whether such an enhancing effect could be found in another inorganic ion, specifically Sr(II), which has been recently reported to affect the morphology of calcite [12].

## 2. Materials and Methods

**Figure 1:**
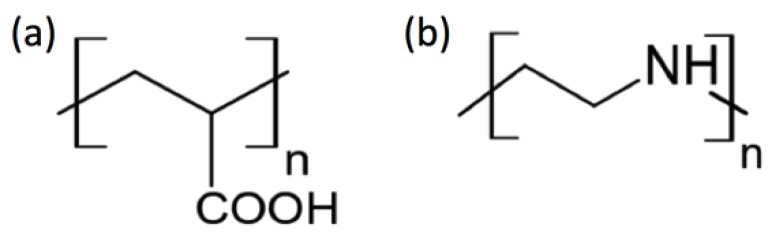
Polymeric additives in the present study: (**a**) poly(acrylic acid) (PAA); (**b**) poly(ethylene imine) (PEI).

All chemicals in the present study were purchased from Sigma-Aldrich (Milwaukee, WI, USA): calcium chloride (CaCl_2_, 97%, anhydrous), ammonium carbonate ((NH_4_)_2_CO_3_, ACS Grade), strontium chloride (SrCl_2_, 99%, hexahydrate), poly(acrylic acid) (PAA, M_w_ (weight average molecular weight, g/mol) 1800), and poly(ethylene imine) (PEI, M_w_ 1300, 50 wt% aqueous solution). The molecular structures of PAA and PEI are shown in Figure 1. Deionized water (resistivity >18.2 MΩ∙cm) was purchased from Direct Q3 (Millipore; Burlington, MA, USA).

Crystallization of calcium carbonate was performed by diffusion of carbon dioxide, generated from ammonium carbonate, into the aqueous solution of calcium chloride [10,11,13]. The solution of calcium chloride (10 mM, 3 mL) was prepared in a 10 mL glass vial at room temperature by completely dissolving the calcium chloride (stirring for 1 h with a magnetic stir bar after capping). Note that the calcium concentration of seawater is also approximately 10 mM [14]. The solution was then moved to a glass petri dish (5 cm diameter), which was sealed with a paraffin film but with a hole (1–2 mm diameter) in the middle for the entry of carbon dioxide. The petri dish was placed in a desiccator (3 L volume), where the ammonium carbonate powder (2.5 g) was placed 5 cm below the dish. After the desiccator was sealed, it was placed in an incubator (BF-150LI, BioFree, Seoul, Korea) at 25 °C. After 48 h, the crystallization was finished by filtering the solution to collect crystals on a polycarbonate track-etched membrane (13 mm diameter, 1 μm pore size; Whatman, Maidstone, UK) and washing lightly with deionized water. The crystals were dried for 24 h at room temperature. The glass petri dish was also lightly washed with deionized water and dried.

When strontium was present, strontium chloride was added to the calcium chloride solution at appropriate concentrations (0.10 mM, 0.20 mM, and 0.40 mM) before placing in the desiccator. Note that the strontium concentration of seawater is about 0.08–0.09 mM [15]. When PAA or PEI was added to the calcium chloride solution, the polymer concentrations were 1 µM and 5 μM. All other procedures were the same as without additives.

Crystal morphology was observed via scanning electron microscopy (SEM, GeminiSEM 300, Zeiss, Oberkochen, Germany). Thin Au coatings were applied using a sputter coater (Q150R S, Quorum, Lewes, UK) to minimize charging. Shape software (version 6.0; Shape Software, Kingsport, TN, USA) was used for the morphological simulation with the known crystallographic information of calcite [16]. Also, crystal phases were analyzed based on X-ray diffraction (XRD, D2 PHASER, Bruker AXS, Billerica, MA, USA) using CuKα radiation (λ = 1.5406 Å) at 30 kV and 10 mA. The 2θ-θ mode was employed to scan a 2θ range of 20–60° at a scanning rate of 1°/min (0.02° increment). A zero-background sample holder (Bruker AXS, Billerica, MA, USA) was used to minimize background noise.

## 3. Results and Discussion

**Figure 2:**
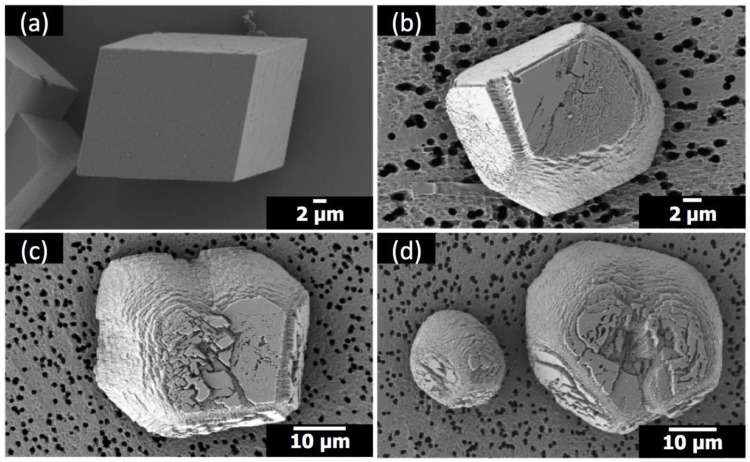
Scanning electron microscopy (SEM) images of the calcite crystals formed with: (**a**) no additives; (**b**) 5 μM poly(acrylic acid) (PAA) at Sr/Ca = 1:100; (**c**) 5 μM PAA at Sr/Ca = 1:50; (**d**) 5 μM PAA at Sr/Ca = 1:25.

**Figure 3:**
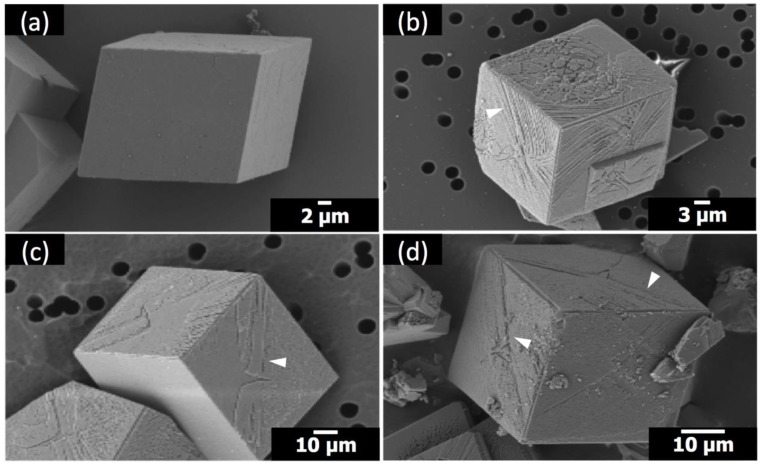
SEM images of the calcite crystals formed with: (**a**) no additives; (**b**) 1 μM PAA at Sr/Ca = 1:100; (**c**) 1 μM PAA at Sr/Ca = 1:50; (**d**) 1 μM PAA at Sr/Ca = 1:25.

The crystals of calcium carbonate obtained in the presence of Sr(II) and PAA are shown in Figure 2 and Figure 3, along with the control experiment (no additive). The crystal morphology with no additive was rhombohedral, enclosed by {104} faces, characteristic of calcite [7,8,9,10]. The molar ratio of Sr/Ca varied at 1:100, 1:50, and 1:25, where Ca(II) concentration was set as 10 mM and Sr(II) concentration was varied 0.10 mM, 0.20 mM, and 0.40 mM, respectively. The concentration of PAA was 5 μM (Figure 2) and 1 μM (Figure 3). In all cases in the present study, the polymorph of calcium carbonate crystals was determined as calcite based on the XRD patterns (Figure S1).

When Sr(II) coexisted with PAA (5 μM) (Figure 2), the acute sides of the calcite crystals were rounded, indicating the formation of new faces other than {104} [7,8]. The extent of morphological alteration was proportional to the amount of Sr(II) (see Figure 2b–d). It was quantified as the ratio of acute/obtuse edge length, which was intrinsically unity for the rhombohedral shape and increased as the acute edges became less linear. The span of obtuse edges was set as the length of the linear perimeter bordering on adjacent {104} faces. The acute/obtuse ratios were 1.27, 1.29, and 1.37 for Sr/Ca = 1:100, 1:50, and 1:25 (5 μM PAA), respectively.

When Sr(II) was with 1 μM PAA, the morphology of calcite did not change as much (Figure 3). The overall rhombohedral shape was preserved in all cases. However, a distinctive surface attribute appeared: striated features (marked with white arrowheads) nearly parallel to the diagonal of the {104} surfaces. This feature was not observed unless both additives were present at the same time as explained in the following paragraphs. This suggests that the diagonally striated features are the combined effects of Sr(II) and PAA. Still, it is yet to be determined whether this feature is possibly a nascent form of the more drastic changes observed with Sr(II) and 5 μM PAA.

**Figure 4:**
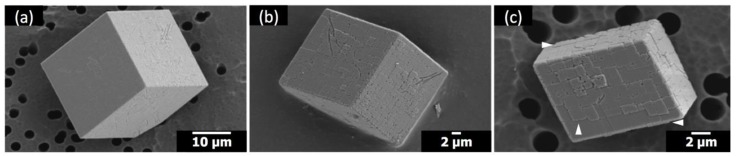
SEM images of the calcite crystals formed with Sr(II) and without polymeric additives: (**a**) Sr/Ca = 1:100; (**b**) Sr/Ca = 1:50; (**c**) Sr/Ca = 1:25.

When either Sr(II) or PAA was present without the other counterpart, the corresponding morphological alterations could not be observed. The calcite morphologies with the addition of only Sr(II) are shown in Figure 4. The overall rhombohedral shapes of calcite were preserved at all concentrations of Sr(II) in the present study. Still, the compartmentalized surface features, whose extent was proportional to the concentration of Sr(II), clearly attested to the growth-disrupting effects of Sr(II) [17]. For the case of Sr/Ca = 1:25 (Figure 4c), subtle protrusion of some compartments from the underlying surfaces was visible (marked with white arrowheads). This observation indicated that Sr(II) hindered the step propagation to localize the crystal growth, which was in accordance with a study of in situ atomic force microscopy on the calcite step growth with the addition of Sr(II) [18]. Alternatively, Sr incorporation in calcite could alter the lattice parameters of the growing crystal layers to induce lattice strain that may lead to the compartmentalized surface features [19]. In fact, the {104} XRD peak was shifted down 0.06° and 0.08° from that of neat calcite for Sr/Ca = 1:100 and 1:25 (Figure S1), respectively, which corresponded to the 2.9 wt% and 3.8 wt% Sr incorporation based on the analysis by Hodkin et al. [12]. Still, the disruption did not generate new types of crystal surfaces, preserving the overall rhombohedral morphology of the crystals. We note here that the crystallization method (carbon dioxide diffusion at pH 6–9) in the present study is quite different from that in the previous report on Sr(II) (homogeneous precipitation at pH > 12 with calcite seed crystals; Sr/Ca ratio up to 20 times higher), making direct comparisons implausible [12].

**Figure 5:**
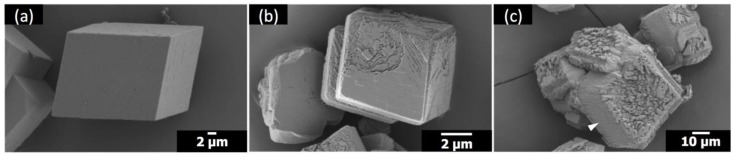
SEM images of the calcite crystals formed with: (**a**) no additives; (**b**) 1 μM PAA without Sr(II); (**c**) 5 μM PAA without Sr(II).

The calcite morphologies with the addition of only PAA are shown in Figure 5. PAA also appeared to disrupt the growth of calcite but in a less regular way than Sr(II), which is consistent with the findings in previous studies [11,20]. With 1 μM PAA, the effect was mainly localized on the {104} surfaces of calcite. As PAA concentration increased to 5 μM, uneven surface features became more predominant, and the emergence of new types of surfaces along the edges between {104} faces became more apparent (Figure 5c, marked with a white arrowhead). Still, the overall rhombohedral features appeared largely preserved. Note that calcite crystals aggregated at the higher PAA concentrations (Figure 5c) without apparent crystallographic preferences, which makes it difficult to ascertain the shapes of individual calcite crystals.

**Figure 6:**
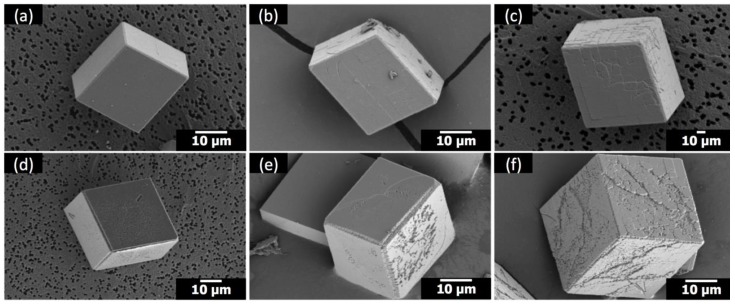
SEM images of the calcite crystals formed with: (**a**) 1 μM poly(ethylene imine) (PEI) (no Sr); (**b**) 1 μM PEI at Sr/Ca = 1:100; (**c**) 1 μM PEI at Sr/Ca = 1:25; (**d**) 5 μM PEI (no Sr); (**e**) 5 μM PEI at Sr/Ca = 1:100; (**f**) 5 μM PEI at Sr/Ca = 1:25.

When Sr(II) was combined with PEI, a cationic polymer at the pH condition (pH 7–9) of the present study, the effects appeared to be additive (Figure 6) compared to the synergistic cooperative effects observed with anionic PAA (Figure 2). When Sr(II) and PEI coexisted, the overall rhombohedral morphology of calcite was preserved (Figure 6), which immediately distinguished the influence of Sr(II)/PEI from that of Sr(II)/PAA. The modification of calcite due to the addition of Sr(II) and PEI was mostly limited to the {104} surfaces. The compartmentalized features showed up at Sr/Ca = 1:100 (Figure 6b,e) and became intensified at Sr/Ca = 1:25 (Figure 6c,f), which was consistent with the morphological changes with the addition of Sr(II) only (Figure 4). The effect of PEI on the {104} surfaces appeared more random without apparent correlation with the crystallographic directions innate to calcite, which was in accordance with the previous study [11]. The effect of PEI appeared somewhat intensified with the addition of Sr(II). This could be related to the increase of the surface edges with the compartmentalized features induced by Sr(II) and/or to the hindered crystal growth by Sr(II), allowing better chances for the PEI molecules to adsorb onto the calcite surfaces.

**Figure 7:**
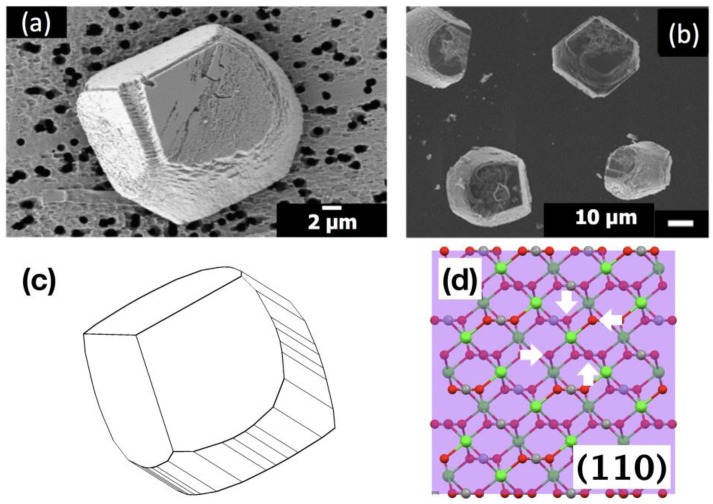
SEM images of the calcite crystals formed with: (**a**) 5 μM PAA at Sr/Ca = 1:100; (**b**) 1 μM PAA at Mg/Ca = 1:10 (reproduced from reference [8]; copyright 2016 The Polymer Society of Korea); (**c**) simulated morphology of calcite with some {hk0} faces in addition to {104}; (**d**) deficient number (4) of oxygen (white arrows) surrounding a calcium on (110) plane (reproduced from reference [7]).

Overall, the cooperative modulation of calcite morphology by Sr(II) and anionic PAA was positively identified. This was the second case of the cooperative effect by inorganic ions and organic macromolecules (Mg(II) and anionic macromolecules being the first such case [10,11]). The modified morphology was quite similar between the two cases (Figure 7a,b). Also, only anionic macromolecules were effective in both cases, indicating the deficient number of oxygens surrounding Ca(II) in new types of surfaces (Figure 7c,d) played a vital role to enable selective binding of anionic molecules to ultimately extend the new surfaces and modify the overall calcite morphology [10,11]. It is interesting to note here that the Sr incorporation, based on the down shift of the {104} XRD peak (Figure S1), was reduced with the addition of PAA (5 μΜ): 2.9 wt% (Δ2θ = 0.06°) to 1.4 wt% (Δ2θ = 0.03°) for Sr/Ca = 1:100 and 3.8 wt% (Δ2θ = 0.08°) to 2.4 wt% (Δ2θ = 0.05°) for Sr/Ca = 1:25 [12]. This could indicate that PAA molecules were adsorbing on the site rich in Sr(II) and displacing Sr(II) while allowing {hk0} faces to be expressed. Further studies would be necessary to verify the interplay between the interactions of Sr(II) and PAA on the surfaces of calcite crystals.

## 4. Conclusions

In summary, the combined effects of Sr(II) and anionic PAA on the morphology of calcite crystals were studied in the concentration range of Sr(II) comparable to the natural abundance (0.08–0.09 mM) in seawater [15]. When Sr(II) coexisted, anionic PAA was able to transform the rhombohedral morphology of calcite, surrounded only by {104} surfaces, into an atypical shape enveloped with new types of surfaces capped with two {104} surfaces when looking down on a {104} face (Figure 7a). This behavior was nearly identical to the cooperative effects of Mg(II) and anionic macromolecules, such as PAA stabilizing {hk0} faces (Figure 7c) [10,11]. The Sr(II) with cationic PEI did not show similar behavior, confirming that the oxygen deficient {hk0} was stabilized through the electrostatic binding of PAA [21,22]. It is of interest to note that the behaviors of anionic macromolecules are comparable when Sr(II) or Mg(II) coexist, both of which are in the natural environment and available during biomineralization. The possibility of a similar cooperative mechanism in play during biomineralization has to be tested with a model crystallization system that mimics the non-classical crystallization of biominerals, especially with amorphous precursors. Experiments are currently in progress to establish a simple crystallization system with a stabilized amorphous calcium carbonate.

## Supplementary Materials

The following is available online at https://www.mdpi.com/1996-1944/12/20/3339/s1, Figure S1: XRD patterns for the representative calcite crystals: (**a**) full spectra (2θ range 20–60°); (**b**) zoomed-in spectra where the positions of {104} peaks marked with blue bars.
