# Supplementary material for: The Combined Effects of Sr(II) and Poly(Acrylic Acid) on the Morphology of Calcite
Source: Materials (Basel). 2019 Oct 13;12(20):3339. doi: 10.3390/ma12203339 (PMC6829221; doi:10.3390/ma12203339)
Supplement: Supplementary file 1 [file materials-12-03339-s001.pdf]

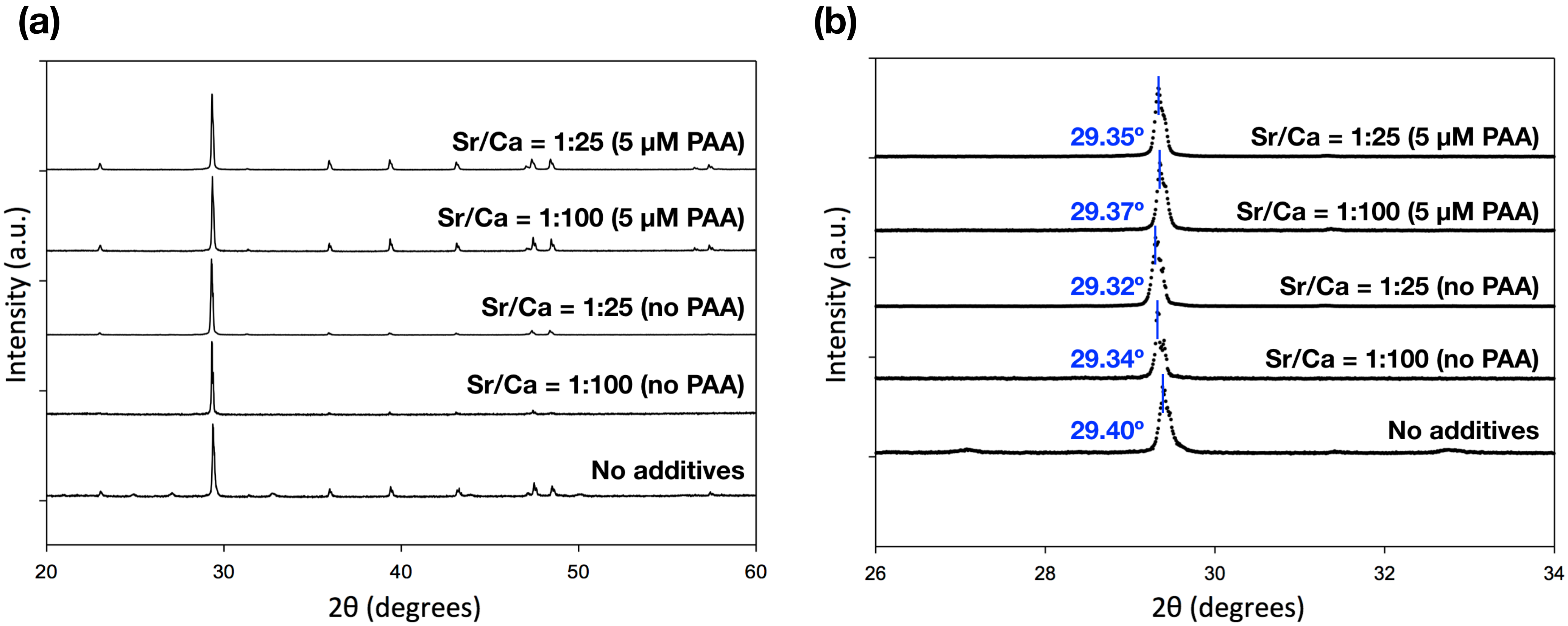

Figure S1. XRD patterns for the representative calcite crystals: (a) full spectra (2θ range 20–60º); (b) zoomed-in spectra where the positions of {104} peaks marked with blue bars.
